# Genetic Characterization of an Avian Influenza Virus H4N6 Strain Isolated from a Guangxi Pockmark Duck

**DOI:** 10.1128/genomeA.00802-17

**Published:** 2017-08-24

**Authors:** Liji Xie, Zhixun Xie, Aiqiong Wu, Sisi Luo, Minxiu Zhang, Li Huang, Zhiqin Xie, Jiaoling Huang, Yanfang Zhang, Tingting Zeng, Xianwen Deng

**Affiliations:** Guangxi Key Laboratory of Veterinary Biotechnology, Guangxi Veterinary Research Institute, Nanning, Guangxi Province, China

## Abstract

An H4N6 subtype avian influenza virus was isolated from a pockmark duck in southern China in November 2013 and named A/duck/Guangxi/149D24/2013 (H4N6). All eight gene segments of the strain were sequenced. Sequence analysis indicated that this H4N6 virus was a natural reassortant virus. This H4N6 virus has two basic amino acids in the cleavage site of hemagglutinin 1 (HA1) and HA2, and the amino acid motif of cleavage site was PEKASRGLF, which is the typical characteristic of the low-pathogenic avian influenza virus. This study will help understand the epidemiology and molecular characteristics of avian influenza virus in pockmark ducks.

## GENOME ANNOUNCEMENT

Avian influenza virus (AIV) is a negative-sense segmented RNA virus that belongs to the genus *Influenza virus A* of the family *Orthomyxoviridae* (1, 2). At present, there are 18 hemagglutinin (HA) and 11 neuraminidase (NA) subtypes of AIV based on the antigenic differences of the HA and NA proteins, which are surface glycoproteins on the viral envelope (3, 4). This H4 subtype of AIV belongs to low-pathogenic AIV (LPAIV), which is one of the predominant subtypes among LPAIV. The H4 subtype of AIV has been circulating and evolving in live poultry markets in China (5). It has been shown that the H4 subtype of AIV has infected migratory water birds and domestic ducks (6, 7). In addition, the H4 subtype of AIV has infected pigs and poses a threat to mammals (8). It may have the ability to cross the species barrier to infect humans through gene reassortment, thus signifying the importance of enhancing the surveillance of the H4 subtype of AIV.

An H4N6 subtype AIV was isolated from a pockmark duck in Guangxi, China, in November 2013 and named A/duck/Guangxi/149D24/2013 (H4N6). All eight gene segments were amplified by reverse transcription-PCR using AIV universal primers (9, 10). The amplified products were gel purified, cloned into the pMD-18T vector (TaKaRa, Dalian, China), and sequenced (TaKaRa). The sequences were assembled using the SeqMan program and manually edited to generate the final full-length genome sequence.

The complete genome of the A/duck/Guangxi/149D24/2013 (H4N6) strain consists of eight gene segments of polymerase basic 2 (PB2), PB1, polymerase acidic (PA), HA, nucleoprotein (NP), NA, matrix (M), and nonstructural (NS) genes. The full lengths of these segments are 2,341, 2,341, 2,233, 1,738, 1,565, 1,464, 1,027, and 890 nucleotides, respectively. The amino acid residues at the cleavage site (positions 338 to 346) of the HA molecule are PEKASRGLF, with two basic amino acids, which is characteristic of low-pathogenic AIV.

Sequence analysis revealed that the nucleotide sequences of the HA and NA genes of the A/duck/Guangxi/149D24/2013 (H4N6) strain both belong to the Eurasian lineage. The nucleotide homology comparisons revealed that the HA gene of this strain shares 99% homology with the HA gene of a Jiangxi AIV strain, A/duck/Jiangxi/32180/2013 (mixed type) (GenBank accession number KP286940). The NA gene shared the highest sequence homology, at 99%, with A/Duck/Thailand/CU-11825C/2011 (H3N6) (GenBank accession number KJ161948). The PB2 gene shared the highest sequence homology, at 96%, with A/wild duck/Korea/SNU50-5/2009 (H5N1) (GenBank accession number JX497765). The PB1 gene shared the highest sequence homology, at 98%, with A/wild duck/Korea/PSC6-1/2009 (H4N6) (GenBank accession number JX454743). The PA and NP genes shared the highest sequence homology, at 99%, with A/Duck/Vietnam/LBM48/2011 (H3N2) (GenBank accession number LC028079). The M gene shared the highest sequence homology, at 98%, with A/duck/Guangxi/GXd-1/2011 (H1N2) (GenBank accession number KF013919). The NS gene shared the highest sequence homology, at 99%, with A/duck/Taiwan/WB459/04 (H6N5) (GenBank accession number DQ376795).

These data indicate that the A/duck/Guangxi/149D24/2013 (H4N6) strain is a novel reassortant virus whose genes derived from multiple AIV strains, and its genome information is useful for analyses of epidemiology and evolutionary characteristics.

### Accession number(s).

The genome sequence of A/duck/Guangxi/149D24/2013 (H4N6) was deposited in GenBank under the accession numbers MF399054 to MF399061.
